# An improved Dijkstra cross-plane image encryption algorithm based on a chaotic system

**DOI:** 10.3389/frai.2024.1394101

**Published:** 2024-06-10

**Authors:** Pijun Hou, Yuepeng Wang, Ziming Shi, Pan Zheng

**Affiliations:** ^1^The Key Laboratory of Advanced Design and Intelligent Computing, School of Software Engineering, Dalian University, Dalian, China; ^2^DHC IT Company, Dalian, China; ^3^Experimental Center, Dalian University, Dalian, China; ^4^Information Systems, University of Canterbury, Christchurch, New Zealand

**Keywords:** cross-plane scrambling, adaptive diffusion, image encryption, chaotic system, Dijkstra algorithm

## Abstract

While encrypting information with color images, most encryption schemes treat color images as three different grayscale planes and encrypt each plane individually. These algorithms produce more duplicated operations and are less efficient because they do not properly account for the link between the various planes of color images. In addressing the issue, we propose a scheme that thoroughly takes into account the relationship between pixels across different planes in color images. First, we introduce a new 1D chaotic system. The performance analysis shows the system has good chaotic randomness. Next, we employ a shortest-path cross-plane scrambling algorithm that utilizes an enhanced Dijkstra algorithm. This algorithm effectively shuffles pixels randomly within each channel of a color image. To accomplish cross-plane diffusion, our approach is then integrated into the adaptive diffusion algorithm. The security analysis and simulation results demonstrate that the approach can tackle the issue of picture loss in telemedicine by encrypting color images without any loss of quality. Furthermore, the images we utilize are suitable for both standard RGB and medical images. They incorporate more secure and highly sensitive keys, robustly withstanding various typical ciphertext analysis attacks. This ensures a reliable solution for encrypting original images.

## 1 Introduction

Image encryption technology is gaining popularity due to its ability to enhance the security of image communication. This is especially crucial as people become increasingly aware of security issues during image transmission (Liang et al., 2022). Image encryption can storage by converting it from significative plaintext into purposeless ciphertext to defend it against permission access and malicious attacks (Huang et al., 2022).

To maintain digital images’ security, researchers have proposed many attack-resistant techniques, including data hiding (Ahmadian and Amirmazlaghani, 2019), image encryption (Hu, 2021; Huang et al., 2022; Li et al., 2023), digital watermarking (Zhang X et al., 2020), and compressive sensing (Wang and Su, 2021; Chai et al., 2022; Sarangi and Pal, 2022). Of such techniques, image encryption is often known for being a direct and significant technique, and utilizing the proper key is the only method to recover the original image data. Over the last several years, a number of approaches have been used to build plenty of digital image encryption algorithms, such as the DNA coding encryption scheme (Liang and Zhu, 2023), the quaternion technique (Wang X. et al., 2022; Wang Y. et al., 2022), and the scheme using block compressive sensing and elementary cellular automata (Chai et al., 2018), it uses cellular automata scrambling to achieve the goal of making pixel values more difficult to predict, and the new zigzag global scrambling scheme designed (Li H et al., 2022). These programs offer multiple benefits and a high level of security.

Chaotic systems have complex dynamic characteristics, unique inherent randomness, control parameters, initial value sensitivity, traversal, and long-term unpredictability, making them appropriate for application in digital image encryption. Andono and Setiadi (2022) introduce several common chaotic systems and utilize multiple multidimensional chaotic systems, such as Lorenz system and Henon map to complete the image encryption. Researchers (Mansouri and Wang, 2020) improved the 2D Arnold mapping by obtaining a scrambled Arnold mapping. Hua et al. (2018) used sine and logistic mappings to produce a new chaotic 2D system. Although this algorithm has high complexity and hyper-chaotic behavior, most multidimensional chaotic systems have high computational costs. In addition, a 1D chaotic system (Wang et al., 2021) was developed and the designed system has the advantages of fast computation and fast image encryption, resulting in time savings.

While color photos are more information-dense than grayscale images, the majority of color image encryption techniques now in use have certain clear shortcomings. The algorithm in Li Q et al. (2022) uses a self-designed inter-plane rule, which requires the calculation of the pixel inter-plane position each time, leading to repeated calculations and a failure to maximize the relationship between pixels and planes. Furthermore, the algorithm in Hua et al. (2021) uses a Latin cube to design a set of scrambling rules for RGB images. For developing the encryption results and safety, the scheme blurs the original image’s pixel values, making the decrypted image inconsistent with the initial image and impossible to fully recover from the initial image. In the later study (Zhou et al., 2021), an RGB image is divided into three planes for independent encryption, and a color image is reconstructed from the encrypted result. In the password system, the security level is low because when a pixel on a plane change, it cannot change quickly enough to extend to three planes. Furthermore, inefficient is this encryption scheme, which ignores the relationship between a color image’s three planes, as a result, real-time encryption systems that demand great security and efficiency are not appropriate for this encryption technique. Images were encrypted using a discrete chaotic system and S-box in the algorithm (Liu et al., 2022), which required over 100 iterations of the S-box and consumed a lot of processing resources. The algorithm (Zheng et al., 2022) use DNA coding to encrypt a portion of the image many times, leading to a poor level of efficiency in the image encryption process.

It is evident from the explanation above that a large number of current encryption techniques for chaotic and color pictures have serious fundamental problems. We provide a fresh approach to color image encryption that makes use of a unique one-dimensional chaotic system for purposed of overcome these problems. The creation of a unique 1D chaotic system with enhanced chaotic performance and a broader parameter range is a main component of this technique. We have developed an improved Dijkstra algorithm that considers the properties of color pictures, building upon the new 1D chaotic system. Rather of encrypting each color plane independently, we accomplish pixel scrambling across color planes. Next, we perform adaptive diffusion based on plane distribution to further alter pixel values and enhance the safety of the encryption method.

The following are this study’s primary contributions:

1. A performance analysis shows that the 1D chaotic system we present eliminates several shortcomings of current chaotic mappings, such as restricted parameters, inadequate nonlinear behavior, and poor unpredictability. According to the analysis of the security performance of Chaos, the new 1D chaotic system proposed by us meets the security requirements, is evenly distributed, and can generate keys that meet the security standards;
2. Many color image encryption techniques have flaws in their architecture. The design of several color picture encryption systems is incorrect. Three distinct gray planes are processed for the majority of color pictures. Using the design of a novel 1D chaotic system and an improved Dijkstra algorithm as the foundation for a cross-plane color encryption technique. Pixels will appear anywhere on any plane, and Adaptive Diffusion Based on Plane Distribution will vary the value of each pixel sufficiently. In contrast to previous color image encryption techniques, our proposed diffusion and permutation operate simultaneously on all three planes, rather than individually on each;
3. Simulation findings and implementation analyses show that our proposed system outperforms several current image encryption techniques in various data aspects and can withstand chosen plaintext attacks.

This essay’s remaining sections are as follows: Chaotic system with a performance study covered in Section 2. The creation of keys and certain encryption procedures, such as diffusion and scrambling methods, are covered in Section 3. Section 4 presents method’s security analysis and simulation findings. Paper’s conclusion is given in Section 5.

## 2 Related work

### 2.1 1D-SASCS chaotic system

1D-SASCS chaotic system (Wang and Liu, 2022) is presented Eq. 1:


(1) $$x_{n+1}=|\sin\left(100\mu /\arcsin x_{n}\right)|$$

λ is a parameter of control, λ∈ (0, +∞). The chaotic system possesses good chaotic characteristics, but the chaotic range of 1D-SASCS is relatively small.

### 2.2 Ill-conditioned matrix

When the data are significantly disrupted, an ill-conditioned matrix exhibits significant oscillations in the solutions of an equation system. Solving linear equation Ax = b, one such matrix is as Eq. 2:


(2) $$\left(\begin{array}{cc} R & 201\\ -800 & 401 \end{array}\right)\left(\begin{array}{c} K_{1}\\ K_{2} \end{array}\right)=\left(\begin{array}{c} 200\\ -200 \end{array}\right)$$

For example, when R = 400, K_1_ = −100, and K_2_ = −200; when R = 402, K_1_ = 99.5025, and K_2_ = 198.01.

### 2.3 1D chaotic system

The formula for 1D chaotic system is as Eq. 3:


(3) $$\left\{ \begin{array}{c} \;\\ \begin{array}{l} A=\left(\begin{array}{c} 400.5+X\left(i\right)-201\\ -800\;401 \end{array}\right)\\ B=\left[\begin{array}{c} 200\\ -200 \end{array}\right]\\ A*\left(\begin{array}{c} K_{1}\\ K_{2} \end{array}\right)=B\\ X\left(i+1\right)=\mathrm{mod}\left(|\sin\left(K_{1}/\arcsin\left(X\left(i\right)\right)\right)+K_{2}|,1\right) \end{array} \end{array}\right.$$

When X(i) ∈ (0, +∞), the mapping demonstrates good chaotic behavior. Compared with certain standard 1D chaotic mappings, our suggested 1D mapping has a wider parameter range. The chaotic system formed when X(1) = 0.5 is adopted by our method, which contains two parameters that vary with each repetition of the X(i) value. Our scheme also widens the chaotic system’s beginning value range.

### 2.4 Diagram of bifurcation

**Figure 1:**
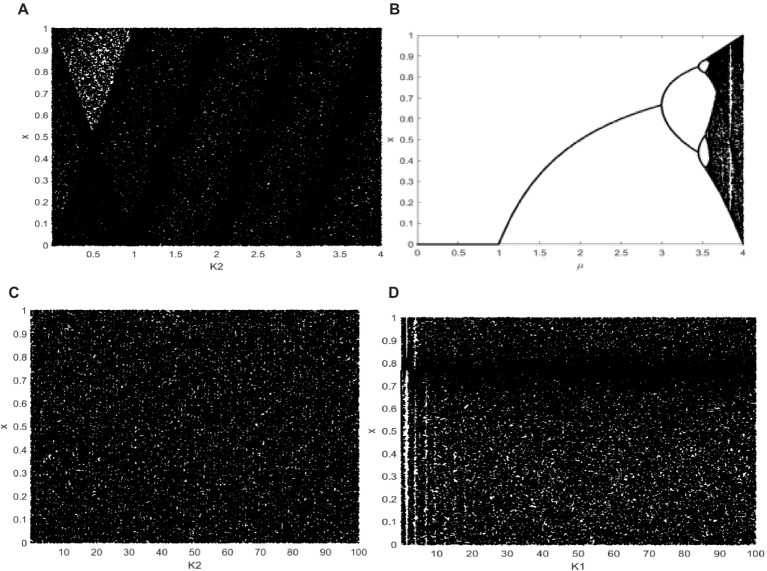
Bifurcation diagram. **(A)** When K_1_ = −465.7689, bifurcation diagram of 1D chaotic map. **(B)** Logistic map, **(C)** When K_1_ = −465.7689, larger range of 1D chaotic map. **(D)** When K_2_ = −465.7689 larger range of 1D chaotic map.

To ensure that the pseudo-random sequence values of chaotic system iteration are evenly distributed throughout a range, bifurcation diagram can be used to visualize the distribution of function values. Figure 1 shows parameter μ range of mapping is represented by the x-axis of the bifurcation diagram, while the values produced by the mapping are represented by the y-axis (Kaçar et al., 2022). One may judge the quality of a chaotic mapping using the bifurcation diagram. 1D chaotic system’s sequence may be examined using the bifurcation diagram to see if it is randomly distributed. In Figure 1A, K_1_ = -465.7689. The logistic mapping bifurcation diagram is displayed in Figure 1B, with parameter μ∈ [0, 4]. In Figures 1C,D, μ∈ [0, 100], K_1_ and K_2_ are set to-465.7689, respectively. The uniform distribution of values within the range of [0,1] is evident, suggesting that the suggested. The chaotic behavior of a 1D system is good. These demonstrate its complicated properties and continuous chaotic range when seen from the perspective of bifurcation trajectory and diagram.

### 2.5 Lyapunov exponent

Lyapunov exponent (LE) is one of crucial reference indices to determining if the chaotic system has especially chaotic qualities. The following formula explains how the LE is Eq. 4:


(4) $$LE=\lim_{n\to \infty }\frac{1}{n}\overset{n-1}{\underset{i=0}{\sum }}\ln|\overset{´}{f}\left(x_{i}\right)|$$

The representation of a chaotic system is f(x_i_). The value of LE may be found in the formula by calculating the derivative of f(x) and averaging the logarithms. A system is considered chaotic when the LE value is higher than 0. Conversely, a system is considered stable when the LE value is smaller than 0. We will determine whether a chaotic system is in a chaotic state within the parameter range by looking at the positive and negative LE values.

**Figure 2:**
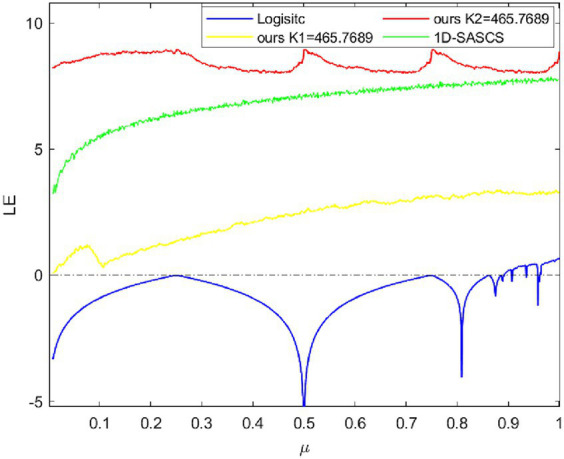
The LE results of logistic map, 1D-SASCS and our method with K_1_ and K_2_.

Figure 2 displays μ∈ (0, 1] LE diagrams for 1D-SASCS chaotic system, the Logistic map and 1D map. We select X(1) = 0.1 in this case, K_1_ = 465.7689 and K_2_ = 465.7689, our chaotic system has a large range of control settings since it consistently maintains a positive LE value. When K_2_ = 465.7689 our LE values are the greatest, suggesting that our chaotic scheme has more complicated nonlinear behavior and superior unpredictability.

### 2.6 Sample entropy

**Figure 3:**
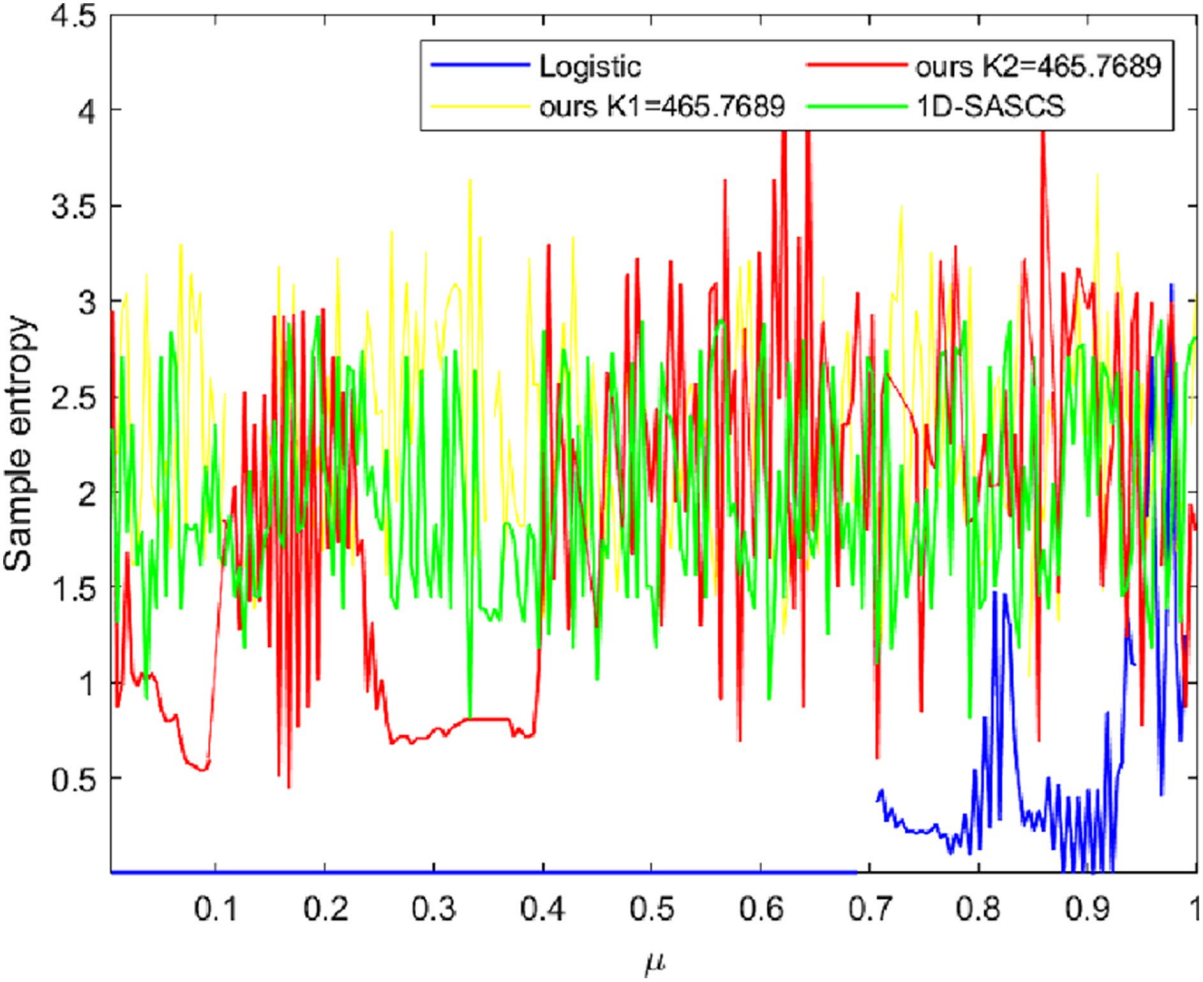
The sample entropy comparison on logistic map 1D-SASCS and our method with K_1_ and K_2_.

The accuracy of sample entropy (SE) (Richman and Moorman, 2000) is higher than that of approximation entropy. The complexity of the output produced by chaotic systems during iteration is measured quantitatively. A positive SE shows chaotic behavior in the created sequence, which deviates from conventional regularity. A higher SE value denotes less regularity in the sequence, which suggests that the chaotic system’s behavior is more complicated. The SE of various chaotic systems is calculated using the computation technique outlined. The SE of our new chaotic system that we have presented is compared with other 1D chaotic systems in Figure 3 and we set the initial value X(1) = 0.5 for all chaos. As can be seen, our suggested 1D chaotic system achieves positive SE values for all control parameters. The outcomes of our experiments show that our chaotic system operates effectively. The computation equations for SE are as Eq. 5:


(5) $$SE\left(m,r,N\right)=-\log\frac{A}{B}$$

In which A and B denote two successive random sequences of chaos, respectively, and m, the array’s dimension, N, the sequence length, and r, the threshold. The Chebyshev distance between A and B is computed, and it is not more than the threshold’s percentage. We set our chaotic, 1D-SASCS and Logistic, X(1) = 0.9, m = 1, r = 0.2. As can be observed, the SE value is somewhat larger than the SE value of other 1D chaos when K_1_ = 465.7689 and is comparatively steady. The SE value is larger than 0 for K_2_ = 465.7689, which satisfies all safety standards.

## 3 Related algorithms

**Figure 4:**
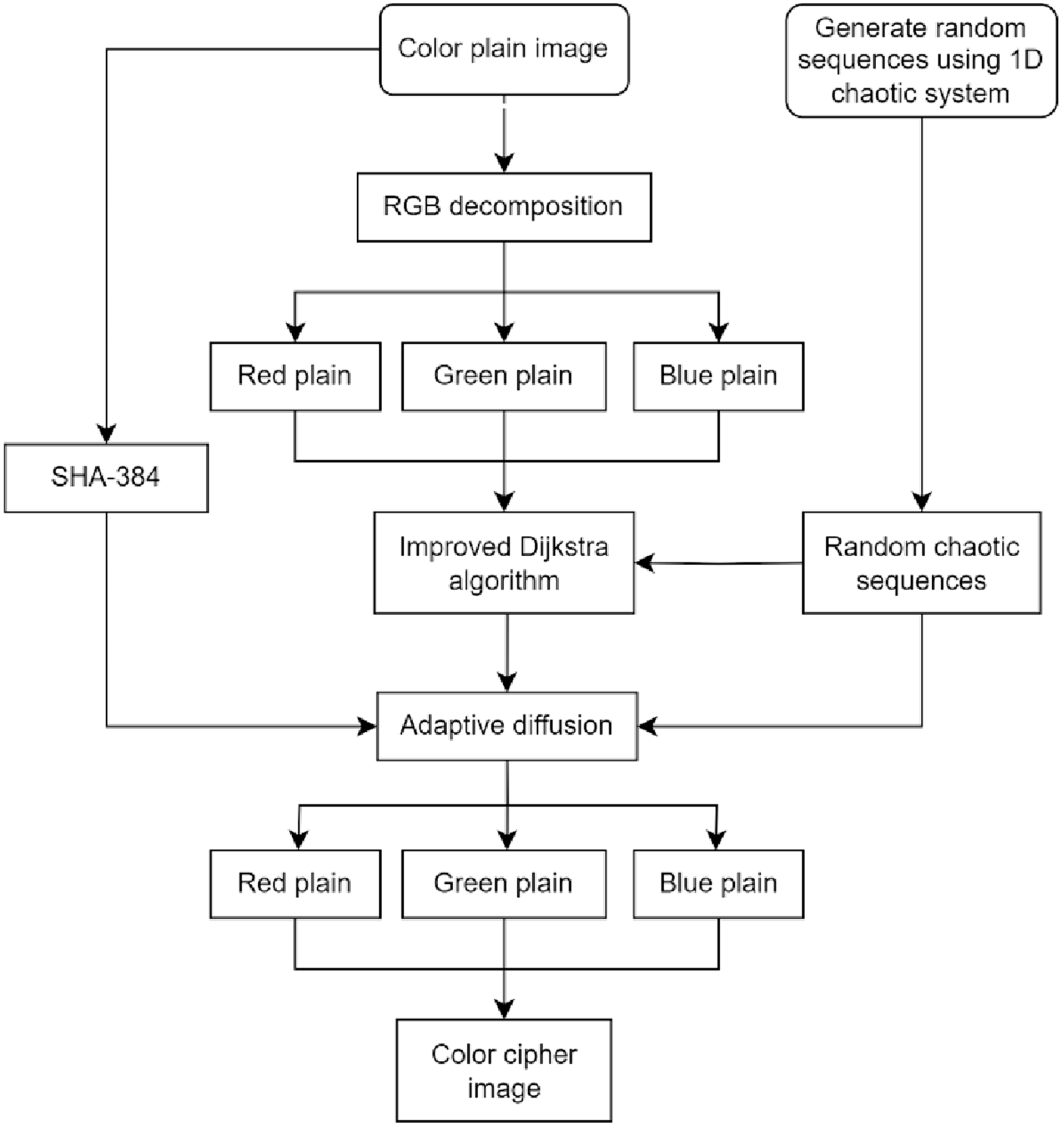
The encryption process for a flowchart.

We introduce a cross-plane color image encryption scheme in the section. The architecture of cross-plane encryption technique is shown in Figure 4. The picture is converted into a 384-bit key using SHA-384. This key and the chaotic matrix produced by a 1D chaotic system are combined to make the encryption key. The image’s three planes are split up, and each plane is simultaneously according to chaotic system and an improved Dijkstra algorithm for cross-plane scrambling. This allows the original image’s pixel to appear at any location in any plane, making it more difficult for an attacker to anticipate where a pixel would appear. An adaptive diffusion approach is used after obtaining the scrambled matrix. This algorithm starts with bidirectional diffusion on the rows and columns, and then moves on to random diffusion over the color planes. Finally, the planes of the image were merged to obtain the final encrypted image. By modifying pixel values to improve security, and because both the improved Dijkstra method and the adaptive diffusion based on plane distribution are reversible, the algorithm can retrieve the original image information using the proper key.

### 3.1 Key generation

The research suggests a key generation process that generates four chaotic sequences using a 1D chaotic system. Because these sequences leverage chaos’ unpredictable nature. To enhance unpredictability, we omit the first 1,000 iterations of the chaotic iterations. Moreover, this key generation mechanism makes ordinary images highly sensitive. The four generated chaotic sequences are denoted as *V_1_*, *V_2_*, *V_3_*, and *V_4_*. *D_1_*, *D_2_*, and *D_3_* are matrices generated from the chaotic sequences, with the size of M × N.

RGB image P to be encrypted is first input into SHA-384 to obtain the 384-bit key Z. Z is the key shifted into 96 decimal numbers, each of which has a length of four digits. Z can be represented as Z = h_1_, h_2_, h_3,_…, h_96_. Next, use Z to obtain parameters *C_1_*…*C_12_*. Then, 3 chaotic sequences of *U_1_*, *U_2_*, and *U_3_* are generated using the specific generation method, as Eqs. 6, 7:


(6) $$\left\{ \begin{array}{c} C_{1}=h_{1}+h_{2}+..+h_{8}\\ C_{2}=h_{9}+h_{10}+..+h_{16}\\ ..\\ C_{12}=h_{89}+h_{90}+..+h_{96} \end{array}\right.$$

(7) $$\left\{\begin{array}{c} U_{1}\left(i,j\right)=\mathrm{floor}\left(\mathrm{mod}\left(\begin{array}{l} V_{1}\left(i,j\right)*10000-\\ \mathrm{floor}\left(V_{1}\left(i,j\right)*10000\right),256 \end{array}\right)\right)\\ U_{2}\left(i,j\right)=\mathrm{floor}\left(\mathrm{mod}\left(\begin{array}{l} V_{2}\left(i,j\right)*10000-\\ \mathrm{floor}\left(V_{2}\left(i,j\right)*10000\right),256 \end{array}\right)\right)\\ U_{3}\left(i,j\right)=\mathrm{floor}\left(\mathrm{mod}\left(\begin{array}{l} V_{3}\left(i,j\right)*10000-\\ \mathrm{floor}\left(V_{3}\left(i,j\right)*10000\right),256 \end{array}\right)\right) \end{array}\right.$$

### 3.2 Dijkstra algorithm

**Figure 5:**
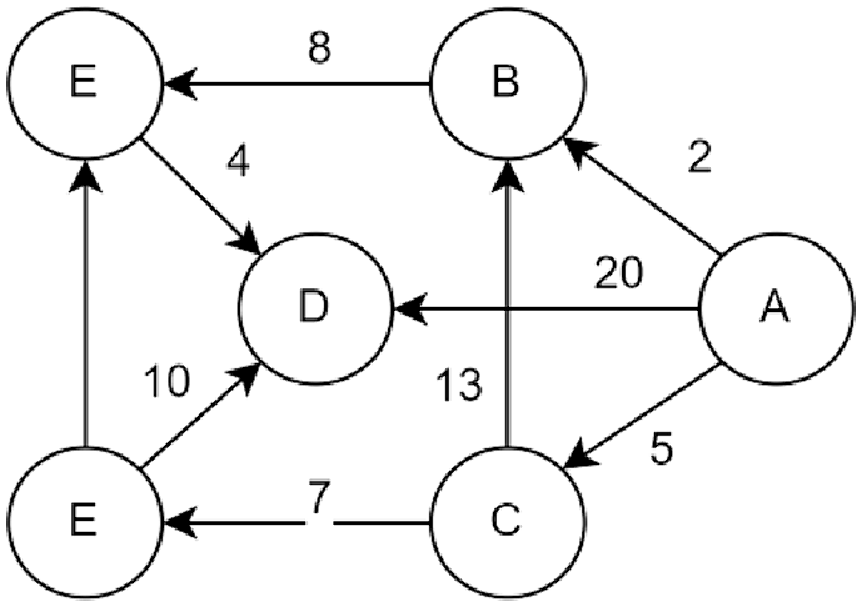
An example of in a directed graph.

One kind of greedy method for determining the shortest path for a single source in weighted networks is the Dijkstra algorithm. It can be applied to both directed and undirected graphs. It is used here to resolve the shortest path issue with directed and undirected graphs. Figure 5 shows that the algorithm starts from vertex A and eventually obtains the set U {A, C, F, B, E, D}.

### 3.3 Improved Dijkstra algorithm

Only pixels on the same plane or multiple operations can be scrambled using conventional color image schemes. Therefore, it is crucial to create a scrambling algorithm that is both effective and secure. This research enhances the position updating procedure to better satisfy the demands of image encryption. As for the pixel weight, which influences both the layer value and the pixel’s coordinates in the plane, we utilize a chaotic matrix. By essentially removing the link between pixel locations and lowering the correlation between neighboring pixels, this method makes it more difficult to anticipate the position of pixels.

Our improved Dijkstra algorithm efficiently makes use of the inter-plane interactions between pixels, shuffle the image pixel position, arrange it across planes, in contrast to conventional color image scrambling techniques. The spatial associations of pixels can be more randomly shuffled, enabling them to appear at random on any plane. This algorithm only requires a single operation to complete the encryption process, rather than encrypting the three planes of an RGB image separately multiple times. It can better leverage the relationships between pixels across different planes, allowing pixels to quickly appear at any position on any plane. Original image P with the size of M*N, this scheme for scrambling H_1_(a, b), H_2_(a, b), and H_3_(a, b) obtained by scanning P from left to right is as shown below.

*Step 1*: The four chaotic sequences *V_1_*, *V_2_*, *V_3_*, and *V_4_* are taken with lengths of M × N, M × N, M × N, and 3 × M × N, respectively.

*Step 2*: Three chaotic matrices are reshaped by processed the *V_1_*, *V_2_*, and *V_3_* chaotic sequences, denoted as *D_1_*, *D_2_*, and *D_3_*, respectively. Where ‘sort’ means to sort the elements of an array. Obtain the index matrices *I_1_*, *I_2_*, and *I_3_* for the three chaotic matrices, as Eq. 8:


(8) $$\left[\sim ,I_{i}\right]=\mathrm{sort}\left(D_{i}\right)$$

*Step 3*: The three planes of image P—*H_1_*, *H_2_*, and *H_3_*—are scrambled to obtain P_1_, P_2_, and P_3_ according to the three index matrices a and b, *D_i_* acts as the pixel’s weight to guide pixel movement, ‘find’ represents a vector that returns a linear index, as Eq. 9:


(9) $$\left\{ \begin{array}{l} \left[m,n\right]=\mathrm{find}\left(D_{i}==N*\left(a-1\right)*b\right)\\ P_{i}\left(m,n\right)=H_{i}\left(a,b\right) \end{array}\right.$$

*Step 4*: Reshape *V_4_* into a matrix with a row length of 3 and a column length of M × N − 1, obtaining matrix *I_4_*. The I_4_ index is sorted by row priority and P1, P2 and P3 are scrambled across planes according to the improved Dijkstra algorithm *I_4_* will guide the pixel to which level of the R, G, B plane, 1, 2, 3 stand for R, G, and B, respectively. Columns’ indicates the plane where the pixel values are located.

**Figure 6:**
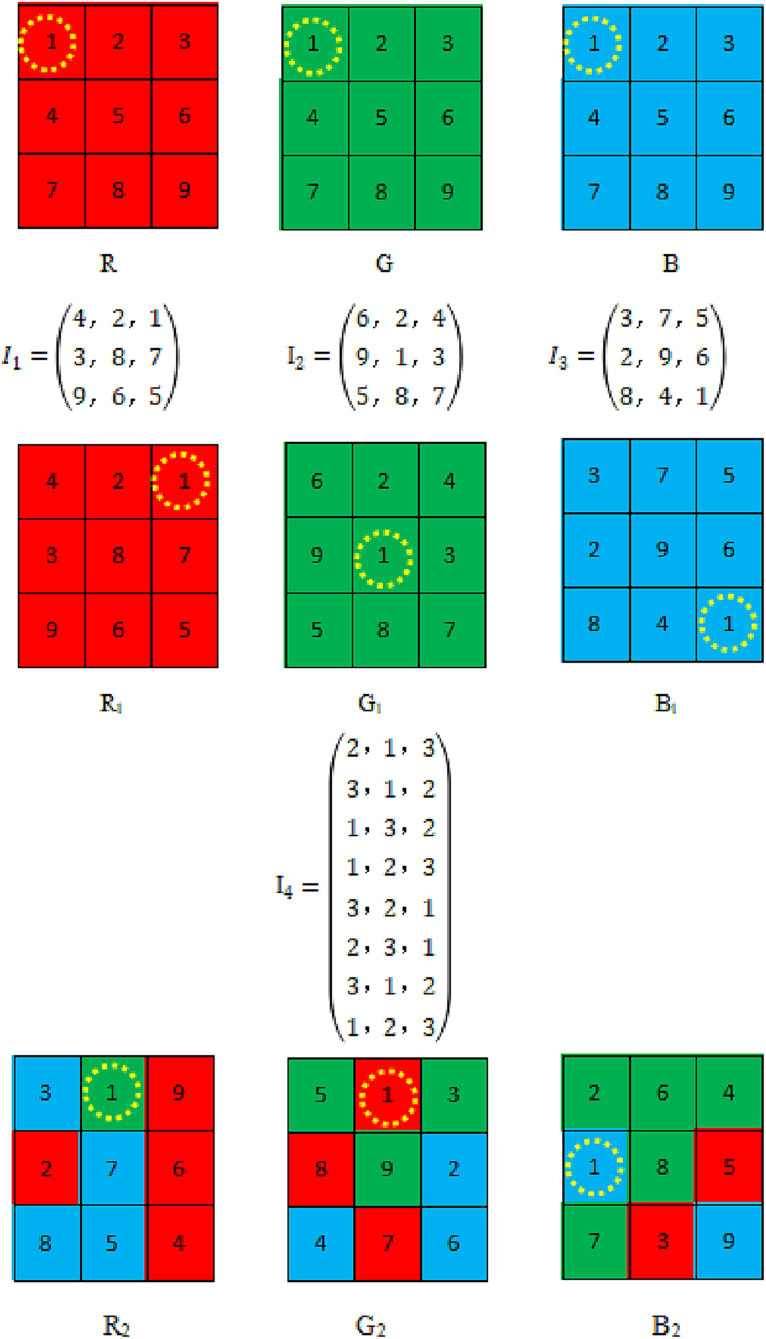
Example of an improved Dijkstra Algorithm.

Both the original Dijkstra algorithm and the improved Dijkstra algorithm are methods for determining the shortest path. The shortest path cross-plane scrambling algorithm is random, and the image pixel is determined by the point-to-point position of the chaotic system, which ensures that each pixel of the image can determine the final position, and ensures the integrity and randomness of the pixel. The magnitude of the comparison weight affects how far pixels shift in relation to their ultimate location. A cross-plane configuration for a 3 × 3 × 3 colored image is shown in Figure 6. Our planes are initially positioned as follows: R(1,1) = 1, G(1,1) = 1, B(1,1) = 1. The positions are changed into R_1_(3,1) = 1, G_1_(2,2) = 1, B_1_(3,3) = 1 based on our input data: *I_1_*(3,1) = 1, *I_2_*(2,2) = 1, *I_3_*(3,3) = 1. This completes the first step of shuffling. The value of *V_4_* specifies the plane into which the pixel will be shuffled, and it indicates the weights allocated to the pathways used to shuffle the image. The row-wise sorting of V_4_ is I_4_. For instance, 2, 1, and 3 are in the first row of *I_4_*. R_1_ = 1 positions are positioned in the second plane, G_1_ = 1 positions are positioned in the first plane, and B_1_ = 1 positions are positioned in the third plane. After guidance, we obtain R_2_(1,2) = 1, G_2_(1,2) = 1, and B_2_(2,1) = 1 based on the index order established: 1 → 2, 2 → 3… →3 × M × N, 3 × M × N → 1. We obtain the final shuffled image when the three planes have finished shuffling. The distribution of each element in the sequence is uniform and random.

### 3.4 Adaptive diffusion based on plane distribution

Encrypted pixels typically solely pertain to the current pixel; they have no effect on following pixels. Even if the current pixel undergoes slight changes in the image. The adaptive diffusion strategy proposed in the paper is based on plane distribution, that is an encryption scheme that utilizes the image’s R, G, and B layers’ pixel values as keys for one another. The image encryption task can be successfully completed with just one diffusion operation on scrambled image. Modifying pixel value of image increases its security and makes it harder for attackers to obtain the original. Specifically, row-column diffusion takes place inside each of the pixels’ individual planes first, followed by diffusion between planes. As a result, pixels differ from one plane to the next. The values of succeeding pixels shift significantly when one does. After the original image has been disturbed by the trans-plane scrambling of improved Dijkstra algorithm. Since neighboring pixels in scrambled image originate from several color planes, The scrambled image is then placed in adaptive diffusion based on plane distribution, the processing sequence is arbitrary and kept a secret, pixel value is severely destroyed by our algorithm, the safety of proliferation is further enhanced. The technique creates a consistent pixel distribution and one-step encryption based on protecting private information, as (Eqs. 10, 11):


(10) $$\left\{\begin{array}{c} R\left(1,j\right)=R\left(1,j\right)⊕U_{1}\left(1,j\right)\\ R\left(i,j\right)=R\left(i-1,j\right)⊕I_{1}\left(1,j\right)⊕R\left(i,j\right)\\ G\left(1,j\right)=G\left(1,j\right)⊕U_{2}\left(1,j\right)\\ G\left(i,j\right)=G\left(i-1,j\right)⊕I_{2}\left(1,j\right)⊕G\left(i,j\right)\\ B\left(1,j\right)=B\left(1,j\right)⊕U_{3}\left(1,j\right)\\ B\left(i,j\right)=B\left(i-1,j\right)⊕I_{3}\left(1,j\right)⊕B\left(i,j\right)\\ R\left(i,1\right)=R\left(i,1\right)⊕U_{1}\left(i,1\right)\\ R\left(i,j\right)=R\left(i,j-1\right)⊕I_{1}\left(i,j\right)⊕R\left(i,j\right)\\ G\left(i,1\right)=G\left(i,1\right)⊕U_{2}\left(i,1\right)\\ G\left(i,j\right)=G\left(i,j-1\right)⊕I_{2}\left(i,j\right)⊕G\left(i,j\right)\\ B\left(i,1\right)=B\left(i,1\right)⊕U_{3}\left(i,1\right)\\ B\left(i,j\right)=B\left(i,j-1\right)⊕I_{3}\left(i,j\right)⊕B\left(i,j\right) \end{array}\right.$$

(11) $$\left\{\begin{array}{c} k=\mathrm{mod}\left(k,12\right)+1\\ R\left(i,j\right)=\mathrm{mod}\left(\left(\mathrm{double}\left(\begin{array}{l} \mathrm{bitxor}\\ \left(\begin{array}{l} R\left(i,j\right),\\ i*j*p\left(k\right) \end{array}\right) \end{array}\right)\right),256\right)\\ G\left(i,j\right)=\mathrm{mod}\left(\left(\mathrm{double}\left(\begin{array}{l} \mathrm{bitxor}\\ \left(\begin{array}{l} G\left(i,j\right),\\ \left(i+j\right).^{2}*R\left(i,j\right) \end{array}\right) \end{array}\right)\right),256\right)\\ B\left(i,j\right)=\mathrm{mod}\left(\left(\mathrm{double}\left(\begin{array}{l} \mathrm{bitxor}\\ \left(\begin{array}{l} B\left(i,j\right),\\ \left(i+j\right).^{2}*G\left(i,j\right) \end{array}\right) \end{array}\right)\right),256\right) \end{array}\right.$$

In this case, M × N represents the encrypted image P’s size. In addition, the image consists of three layers: R, G, and B. The modulo operation is denoted by ‘mod. the bitwise XOR operation by ‘bitxor. and the key generation set in Section 2 is denoted by p(k), where k ranges from 1 to 12.

## 4 Simulation results and security analysis

To address the requirements of many situations, we discuss the results of simulations using a variety of image formats. We also detail a significant amount of security research to show the safety and effectiveness of our approach. All experiments are conducted and simulated using MATLAB 2021a on the laptop with an i7-10710 U CPU. In this paper, two sets of ablation experiments are set up, when the encryption algorithm is only named EX1 using the improved Dijkstra algorithm, and when the encryption algorithm is only used adaptive diffusion based on plane distribution, it is named EX2.

**Figure 7:**
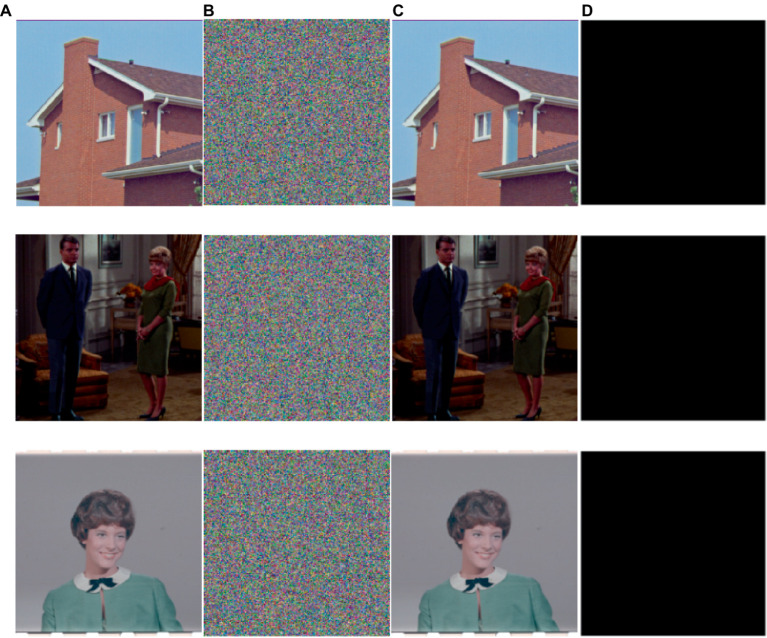
Simulation results of the image encryption algorithm proposed are as follows: **(A)** and **(C)** Initial and decrypted images, **(B)** Encrypted images, and **(D)** Difference between initial images and decrypted image **(A–C)**. The images ‘House’, ‘Couple’ and ‘Female’ have been downloaded from USC-SIPI Image Database.

This part shows the simulation and testing of the encryption technique provided in part 3. We perform tests on the original images by employing different-sized standard test images and using the encryption method suggested in this study as Figure 7 shown. No meaningful data are present in the encrypted image in Figure 7A. The contrast between original image and encrypted image, the latter of which is a completely black image, is also shown in Figure 7D. This result indicates that our method applies to image encryption and retrieves images without any loss.

### 4.1 Simulation results and histogram analysis

**Figure 8:**
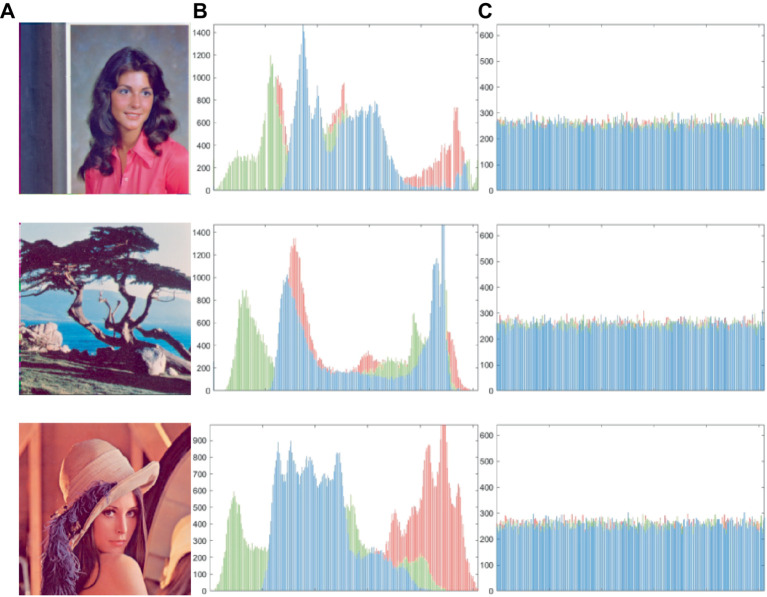
**(A)** The plaintext image, **(B)** and **(C)** the histogram of plaintext and cipher images. The images ‘Female’, ‘Lena’ and ‘Tree’ have been downloaded from USC-SIPI Image Database.

Histogram analysis is a highly effective means of presenting data in a cryptographic system because it provides a visual presentation of the statistical information contained within image pixels. Regarding cryptography, the distribution of the cipher in the histogram must be as uniform as possible because any deviations can provide attackers with valuable statistical information that can be used to compromise the system’s security. As seen in Figure 8, we compared different images using histograms. Figures 8B,C show histograms of plaintext and ciphertext, respectively, demonstrating that our encryption scheme produces a relatively flat histogram. This result suggests that there is some degree of assault resistance in our design.

### 4.2 Key space analysis

A wider key area is necessary to successfully deter attackers from acquiring the correct key. A secure cryptographic system (Chai et al., 2017) often requires a key space greater than 2^100^. Key space for SHA-384 of the scheme is 2^192^ and has 12 keys, which is far greater than 2^100^ to effectively fend against brute-force attack.

### 4.3 Information entropy

A signal source for distribution can be quantitatively described using information entropy. Moreover, 8 is optimal value of entropy for an image of an 8-bit binary. The formula for computing information entropy as Eq. 12:


(12) $$E\left(a\right)=-\overset{255}{\underset{i=0}{\sum }}Q\left(a_{i}\right)\log_{2}p\left(a_{i}\right)$$

Where a_i_ represents a pixel’s value, and *Q*(*a_i_*) stands for the frequency of a_i_. When each value is equally likely to occur, the maximum value will be reached by information entropy. In an 8-bit image, 256 is gray level, and when each pixel appears with a probability of 1/256, the maximum information entropy can be obtained. In this section, we present entropy testing on the Lena (256 × 256 × 3) image, and Table 1 provide a comparison of the test data utilized to develop our approach. Even if our values are not the highest, they are similar and adhere to security standards, which shows our scheme has good performance.

**Table 1:** Information entropy compares the ‘Lena’ (256 × 256 × 3) image with other schemes.

Encryption schemes	R	G	B	Avg
Lena	7.2353	7.5683	6.9176	7.2404
proposed	7.9969	7.9974	7.9970	7.9971
EX1	7.7253	7.7305	7.7292	7.7283
EX2	7.7974	7.7973	7.7972	7.7973
Zhang Y. Q. et al. (2020)	7.9917	7.9912	7.9917	7.9915
Wang X. et al. (2022) and Wang Y. et al. (2022)	7.9973	7.9971	7.9971	7.9972
(Chai et al. (2019)	7.9973	7.9969	7.9971	7.9971
(Hosny et al. (2021)	7.9956	7.9949	7.9953	7.9953

Table 2 shows our scheme results about the entropy for diverse image. Because neighboring pixels are associated, rather than random, the plaintext image has a low entropy. This shows the validity of our hypothesis and comes close to the predicted maximum value of 8. The encrypted images suggested in this paper have erratic distributional properties, from which no usable data can be derived. Table 3 shows the information entropy ablation experiment, and it can be seen that the EX1 and EX2 values are low and do not meet the safety criteria.

**Table 2:** Information entropy of different size and different images.

Image size	Images	Plain images			Cipher images		
		R	G	B	R	G	B
256 × 256 × 3	4.1.01	6.4200	6.4457	6.3807	7.9971	7.9972	7.9967
	4.1.02	6.2499	5.9642	5.9309	7.9971	7.9973	7.9961
	4.1.03	5.7150	5.3738	5.7117	7.9970	7.9973	7.9972
	4.1.04	7.2549	7.2704	6.7825	7.9974	7.9973	7.9974
	4.1.05	6.4311	6.5389	6.2320	7.9977	7.9969	7.9975
	4.1.06	7.2104	7.4136	6.9207	7.9970	7.9971	7.9973
	4.1.07	5.2626	5.6947	6.5464	7.9972	7.9970	7.9971
512 × 512 × 3	4.2.05	6.7178	6.7990	6.2138	7.9993	7.9994	7.9993
	4.2.06	7.3124	7.6429	7.2136	7.9993	7.9992	7.9994
	4.2.07	7.3255	7.3912	6.9169	7.9993	7.9993	7.9994

**Table 3:** Different images of different images of ablation experiments have different sizes of information entropy.

Image size	Images	EX1			EX2		
		R	G	B	R	G	B
256 × 256 × 3	4.1.01	7.7972	7.797	7.7971	7.8972	7.8970	7.8971
	4.1.02	6.2948	6.2921	6.2927	7.8970	7.8973	7.8971
	4.1.03	5.9691	5.9628	5.9749	7.8976	7.8973	7.8975
	4.1.04	7.4229	7.4276	7.4255	7.8970	7.8973	7.8971
	4.1.05	7.0711	7.0615	7.0676	7.8972	7.8970	7.8971
	4.1.06	7.5335	7.5341	7.5377	7.8967	7.8971	7.8969
	4.1.07	6.5855	6.5814	6.5797	7.8972	7.8975	7.8972
512 × 512 × 3	4.2.05	6.6623	6.6639	6.6642	7.9974	7.9973	7.9972
	4.2.06	7.7605	7.7613	7.7632	7.9972	7.9970	7.9971
	4.2.07	7.5839	7.5824	7.5820	7.9893	7.9893	7.9893

### 4.4 Analysis of adjacent pixel correlation

The initial pixels’ regular distribution usually creates a stable correlation between them, which can negatively impact the quality of the ciphertext when introduced in encryption. For evaluating the correlation between relevance pixels in our proposed encryption system, for test items, we select 3,000 pairs of pixels with the formula expressed as Eq. 13:


(13) $$C\left(x,y\right)=\frac{\frac{\sum_{i=1}^{N}\left(X_{i}-L\left(a\right)\right)\left(Y_{i}-L\left(b\right)\right)}{N}}{\sqrt{\frac{1}{N}\sum_{i=1}^{N}{\left(X_{i}-L\left(a\right)\right)}^{2}}\sqrt{\frac{1}{N}\sum_{i=1}^{N}{\left(Y_{i}-L\left(b\right)\right)}^{2}}}$$

Where *L(A)* and *L(B)* are the sequences ‘a’ and ‘b. respectively, in mathematical expectations. A greater correlation between the sequences ‘a’ and ‘b’ is indicated by a larger correlation coefficient, while a correlation coefficient that is closer to zero suggests less correlation.

**Figure 9:**
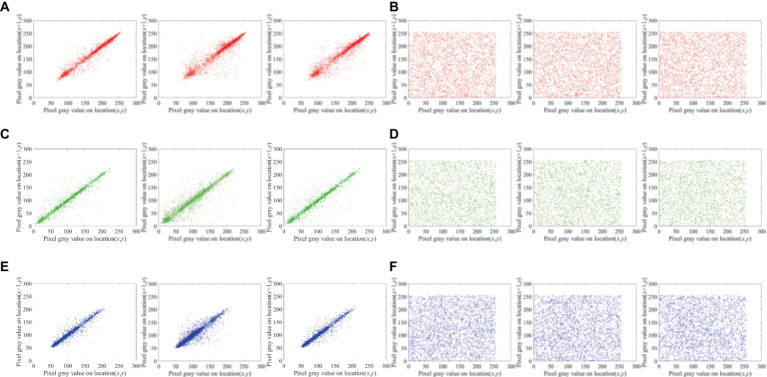
Lena (256 × 256 × 3 px) horizontal, diagonal and vertical distribution of adjacent pixels: **(A)** and **(B)** Distribution of adjacent red pixels in the plaintext and ciphertext, **(C)** and **(D)** distribution of adjacent green pixels in the plaintext and ciphertext, and **(E)** and **(F)** distribution of adjacent blue pixels in plaintext and ciphertext.

In contrast to the accompanying ciphertext image, which is evenly scattered over the plane in a diagonal orientation. Figure 9 displays the pixel distribution in the test image and its surroundings. In Table 4, the correlation coefficients are displayed.

**Table 4:** Correlation among adjacent pixels in different sizes and different images.

Images	Directions	Plain images			Cipher images		
		R	G	B	R	G	B
4.1.01	H	0.9593	0.9678	0.9462	−0.0051	−0.0033	−0.0028
	D	0.9476	0.9563	0.9398	−0.0056	0.0006	−0.0064
	V	0.9766	0.9715	0.9585	−0.0068	−0.0068	−0.0057
4.1.02	H	0.9610	0.9511	0.9491	0.0029	−0.0015	0.0019
	D	0.9167	0.9049	0.8844	0.0062	0.0031	0.0009
	V	0.9588	0.9320	0.9092	0.0079	0.0072	−0.0086
4.1.03	H	0.9453	0.9226	0.8936	0.0060	0.0061	0.0064
	D	0.9125	0.9066	0.8705	−0.0071	0.0008	0.0007
	V	0.9739	0.9752	0.9711	0.0068	−0.0067	0.0049
4.2.05	H	0.9537	0.9678	0.9237	−0.0061	0.0046	−0.0025
	D	0.9354	0.9287	0.9123	−0.0031	−0.0026	0.0041
	V	0.9720	0.9560	0.9648	−0.0035	−0.0053	0.0066
4.2.06	H	0.9576	0.9707	0.9665	−0.0056	−0.0003	0.0026
	D	0.9417	0.9506	0.9515	−0.0015	−0.0005	−0.0056
	V	0.9568	0.9720	0.9731	0.0062	−0.0036	0.0006
4.2.07	H	0.9656	0.9781	0.9671	0.0046	−0.0028	0.0057
	D	0.9533	0.9712	0.9573	−0.0024	−0.0033	0.0045
	V	0.9605	0.9816	0.9628	−0.0057	0.0056	−0.0009

Between proposed scheme and the corresponding ciphertext images of different plaintext images, owing to the large data redundancy of the plaintext image, the nearby pixels exhibit a high correlation coefficient. Since the ciphertext image’s correlation coefficient is practically 0, the suggested approach can be successful in eliminating the substantial relationship between adjacent pixels in plaintext image.

Here, we investigate correlation coefficients of ciphertext images using various encryption techniques. Three planes of the test image Lena, which has dimensions of 256 × 256 px, are used to determine correlation coefficients. Table 5 displays data for the correlation coefficient comparison of various ciphertext images. The values of our scheme are closer to zero, EX1 and EX2 have high correlation between adjacent pixels.

**Table 5:** Comparison of correlation coefficients with different methods using the image ‘Lena’.

Planes	Directions	Plane image	Our scheme	EX1	EX2	Hosny et al. (2021)	Hosny et al. (2022)	Zheng et al. (2023)
R	H	0.9746	−0.0064	0.0150	0.0055	0.0064	−0.0154	0.0071
	D	0.9406	−0.0007	0.0114	0.0119	−0.0026	0.0159	−0.0006
	V	0.9558	0.0039	0.0120	−0.3333	0.0160	−0.0102	0.0089
G	H	0.9722	0.0013	0.0070	−0.0046	0.0009	−0.0096	−0.0012
	D	0.9102	0.0015	−0.0316	0.4410	0.0125	−0.0162	−0.0043
	V	0.9458	0.0045	−0.0101	−0.0371	0.0034	0.0027	−0.0018
B	H	0.9478	0.0030	−0.0213	0.0314	0.0091	−0.0030	−0.0015
	D	0.8776	0.0017	0.0204	−0.0092	−0.0090	−0.0026	−0.0019
	V	0.9318	−0.0063	0.0119	−0.0056	−0.0045	0.0117	0.0041

### 4.5 Differential attack experiment

Differential attack is a extensive used and powerful attack strategy. By evaluating the impact of the change rate of each pixel between original and encrypted images, we find that the best performance indicators for judging differentiated attacks are the number of pixels change rate (NPCR) and unified average changed intensity (UACI). *K_1_* and *K_2_* are two encrypted outputs of the same plaintext image produced after fine-tuning, NPCR and UACI (Gao et al., 2022) calculated as Eqs. 14, 15:


(14) $$\mathrm{NPCR}\left(C_{1},C_{2}\right)=\overset{M}{\underset{i=1}{\sum }}\overset{N}{\underset{j=1}{\sum }}\frac{U\left(i,j\right)}{D}\times 100\%\text{,}$$

(15) $$\mathrm{UACI}\left(C_{1},C_{2}\right)=\overset{M}{\underset{i=1}{\sum }}\overset{N}{\underset{j=1}{\sum }}\frac{\left|K_{1}\left(i,j\right)-K_{2}\left(i,j\right)\right|}{D*F}\times 100\%\text{,}$$

U is the difference between *K_1_* and *K_2_*, *F* is the greatest pixel value, *D* is a total number of color plane pixels. *K_1_*(i, j) = *K_2_*(i, j) if U (i, j) = 0; otherwise, U(i, j) = 1. As shown in Table 6, we perform a comparison test of our method Ex1 and EX2 against others. Using a Lena image (256 × 256 × 3 px). The NPCR and UACI are found to be extremely near to the theoretical maximums of 99.61 and 33.46%, respectively (Kumar et al., 2018). We also observe that our UACI values meet the safety standards and that the NPCR values are higher than those of other methods. EX1 and EX2 does not meet safety standards. Table 7 shows our scheme’s NPCR and UACI values for various image sizes are near the theoretical value, demonstrating the system’s strong potential for differential protection.

**Table 6:** NPCR and UACI data testing using image of ‘Lena’.

Lena	NPCR				UACI			
	R	G	B	Avg	R	G	B	Avg
Ours	99.6323	99.6338	99.6124	99.6261	33.5163	33.4215	33.4666	33.4681
EX1	3.3707	3.3707	3.3707	3.3707	1.8079	1.7963	1.7838	1.7960
EX2	96.7209	82.5531	63.6673	80.9804	32.9611	29.0607	22.1373	28.0530
Hosny et al. (2022)	99.6017	99.6124	99.6368	99.6149	33.4128	33.4980	33.4974	33.4694
Hosny et al. (2021)	99.6094	99.6124	99.6307	99.6175	33.4666	33.4241	33.4212	33.4373
Gao et al. (2022)	99.6180	99.6376	99.6003	99.6189	33.4285	33.4549	33.4275	33.4399

**Table 7:** NPCR and UACI of different images and different planes.

Images	NPCR (%)				UACI (%)			
	R	G	B	Avg	R	G	B	Avg
4.1.01	99.6567	99.5972	99.5697	99.6078	33.4495	33.5133	33.5127	33.4918
4.1.02	99.6216	99.6155	99.6140	99.6170	33.5080	33.5635	33.6437	33.5717
4.1.03	99.5911	99.5850	99.5865	99.5875	33.5879	33.4915	33.4298	33.5030
4.1.04	99.6231	99.6017	99.6048	99.6098	33.4754	33.5302	33.5021	33.5025
4.2.05	99.6181	99.6117	99.6193	99.6163	33.4725	33.5218	33.4894	33.4945
4.2.06	99.6151	99.6155	99.6014	99.6106	33.4426	33.4549	33.4847	33.4607
4.2.07	99.6231	99.5914	99.6220	99.6121	33.5338	33.4807	33.4993	33.5046

### 4.6 Resistance to data loss and noise

**Figure 10:**
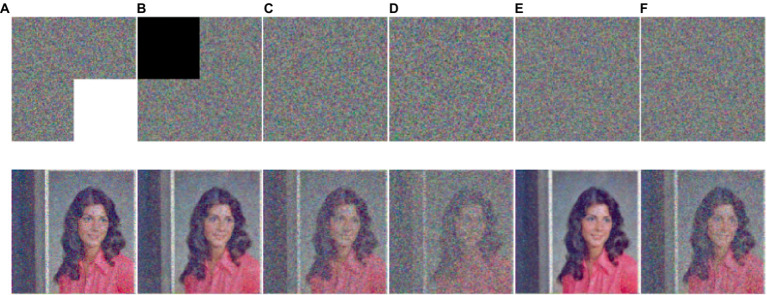
The first row shows the cipher images with data loss and different levels of noise, respectively, **(A)** is missing in the bottom right corner, **(B)** is missing in the top left corner, **(C)** is 0.1 density of salt and pepper noise, **(D)** is 0.2 density of salt and pepper noise, **(E)** is 0.000001 density of Gaussian noise, and **(F)** is 0.000002 density of Gaussian noise, While the second row shows the matching decrypted data. The image ‘Female’ has been downloaded from USC-SIPI Image Database.

The risk of data loss or noise contamination exists while sending data over the internet. Images that have lost data or are tainted by noise must be able to retrieve most of their information when using a trustworthy encryption technique. To evaluate our system’s resistance to these dangers, we simulate data loss and noise pollution on ciphertext image. As shown in Figure 10, we tested different attacks, the experiment proved our method successfully retrieves most of the information while reconstructing an ordinary, visually clear image. Our suggested system can therefore successfully withstand data loss and noise pollution.

Peak signal-to-noise ratio (PSNR), a statistic measures the degree of visual distortion, is objective. When the PSNR is high, we might get results that are closer to original image. The computation equations for PSNR and MSE are as Eqs. 16, 17:


(16) $$\mathrm{PSNR}=10\times \log_{2}\left(\frac{MAX_{I}^{2}}{MSE}\right)\text{,}$$

(17) $$MSE=\frac{1}{A*B}\overset{A-1}{\underset{i=0}{\sum }}\overset{B-1}{\underset{j=0}{\sum }}{\left[M\left(i,j\right)-N\left(i,j\right)\right]}^{2}\text{,}$$

For plaintext and ciphertext images, *M*(i,j) and *N*(i,j) are the values of pixel, respectively. Maximum pixel value for images is *MAX_I_*. Table 8 shows PSNR values larger than 10 dB this technique outperforms previous attack techniques in terms of resistance to Gaussian noise. We may thus draw the conclusion that this plan can ensure security and maintain a strong connection to typical images.

**Table 8:** MSE and PSNR are compared under different attack data.

Cipher image	MSE			PSNR (dB)		
	Red	Green	Blue	Red	Green	Blue
1/4 Data loss at the bottom-right corner	5,415	5,402	5,459	10.7945	10.8045	10.7591
1/4 Data loss at the top-left corner	5,456	5,445	5,402	10.7616	10.7705	10.8046
Gaussian noise = 0.000001	0.0493	0.0497	0.0492	61.1982	61.1701	61.2103
Gaussian noise = 0.000003	0.2601	0.2579	0.2575	53.9789	54.0165	54.0232
Salt&Pepper noise = 0.1	2,207	2,131	2,146	14.6927	14.8440	14.8135
Salt&Pepper noise = 0.2	4,309	4,432	4,365	11.7861	11.6647	11.7309

### 4.7 Image autocorrelation test

2D image autocorrelation compares all possible pairs of two pixels which shows likelihood of having similar values based on distance and separation direction. Generally, the autocorrelation of a planar image is visualized as a wave and cone shape in the spatial domain, whereas the autocorrelation of a cipher image appears as a uniform and level surface. Equation is used for the image autocorrelation is calculated as in Eq. 18:


(18) $$\delta \left(x,y\right)=D^{-1}D\left[O\left(M,N\right)\right]*\bar{D}\left[O\left(M,N\right)\right]$$

**Figure 11:**
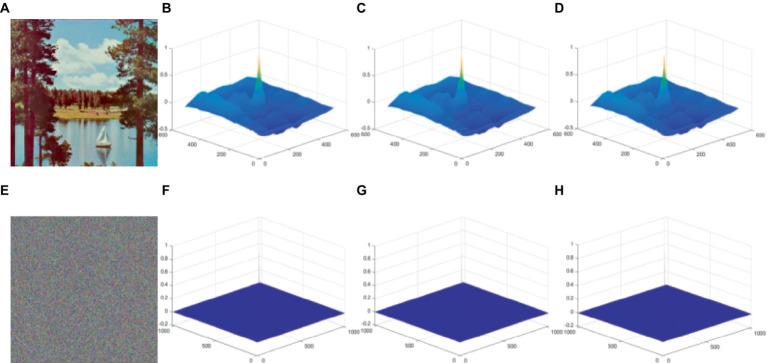
Test for graphic autocorrelation. **(A)** is the original image and **(E)** is the corresponding decrypted image. Plaintext images in the R, G, B planes are subjected to a 3D graphic autocorrelation test for **(B–D)**, and **(F–H)** ciphertext image 3D graphic autocorrelation test in R, G, B planes. The image ‘Sailboat on lake’ has been downloaded from USC-SIPI Image Database.

In this case, *D*^−1^ stands for the conjugate Fourier transform, *O(M, N)* is pixel’s value at position (M, N) in picture, *D* is the Fourier transform, and *P* (x, y) is the autocorrelation function. According to Figure 11, we utilize ‘Tree’ as the test image with an encrypted image across the R, G, B color channels using it as our benchmark. The figure depicts our experimental results. The autocorrelation of the planar image shown in Figures 11B–D demonstrates a wave-like pattern, indicating that the probability of pixel pairs with the same pixel value is higher in planar images. By contrast, the cipher image is smoother according to the test results of autocorrelation (Figures 11F–H), reflecting that our proposed method effectively reduces the probability of equal pixel values.

### 4.8 Floating frequency test

The plain image should uniformly encrypt all rows and columns using a good image encryption technique. A key indicator for assessing an encryption method that can generate stochastic data for all rows and columns and analyze the vulnerabilities in the encrypted image is the floating frequency test (Murillo-Escobar et al., 2019). For example, below is the procedure for determining the row and column floating frequencies for a 256 × 256-px image.

*Step 1*: Set the 256-element image as a window in each row and column.

*Step 2*: Count the number of diverse components in every window.

*Step 3*: Determine a number of different items in each window, as well as the row and column floating frequency values.

*Step 4*: Determine the average values of the floating frequency for rows and columns.

**Figure 12:**
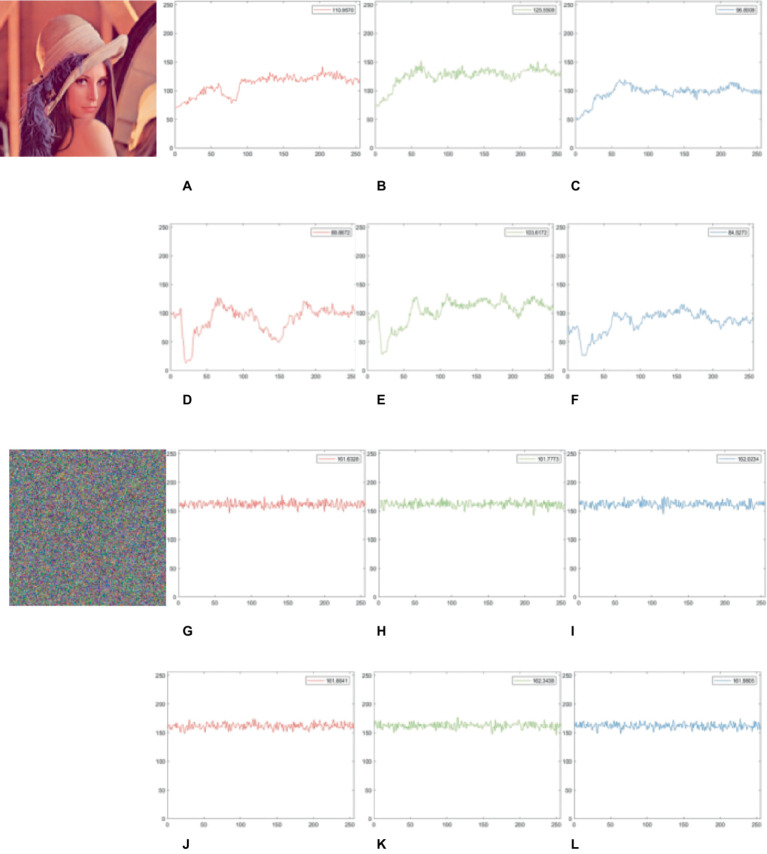
Floating frequency test for plain and cipher images. **(A–C)** and **(G–I)** show the row floating frequency of the plain and cipher image ‘Lena’ in the R, G, B channels. **(D–F)** and **(J–L)** show the column floating frequency of the plain and cipher image ‘Lena’ in R, G, B channels. The image ‘Lena’ have been downloaded from USC-SIPI Image Database.

Here is a sample of the selected color image ‘Lena’. The frequency float test as shown in Figure 12, the row and column floating frequency values for the original image are relatively low (Figures 12A–F), indicating the plain image’s pixel distribution is uneven (with numerous repeated elements). Figures 12G–L displays the cipher image’s row and column floating frequency values, both of which are rather high, at about 161, indicating that nearly 63% of the 256 elements in each column and row are unique. This implies our scheme generates a cipher image and a more uniform component distribution.

### 4.9 *χ*^2^ test

χ^2^ test provi des a quantitative analysis of the homogeneity of the image pixel distribution. We calculate the image’s *χ*^2^ value (Liu et al., 2023) g formula and compare it with the benchmark value. The distribution of the image’s pixels seems to be more uniform when the calculated value is lower than the standard value as Eqs. 19, 20:


(19) $$\chi 2=\overset{255}{\underset{i=0}{\sum }}\frac{{\left(p_{i}-p\right)}^{2}}{f}$$

(20) $$p=\frac{M*N}{256}$$

Where the appearance’s pixel frequency value i in image is represented by *p_i_*, and the average frequency is represented by *p*. The benchmark for ciphertext images in the second test of this technique is 293.24783. As demonstrated in Table 9, the outcomes of this approach for numerous images are provided, and our ciphertext is fairly evenly distributed. EX1 does not meet safety standards, EX2 meets the safety standards but has a higher value than the scenario in this article.

**Table 9:** *χ*^2^ test between different images and planes.

Image	*χ*^2^ test			
	Plain	Cipher	EX1	EX2
4.1.04	81,482	237.3906	46396.2734	259.9679
4.1.05	317,260	238.7396	108529.8567	260.8698
4.1.06	89,401	260.8932	45279.6093	279.6484
4.1.07	486,578	261.7838	199795.3515	264.3880
4.2.05	822,925	249.7903	770085.6176	267.7802
4.2.06	223,807	247.9980	74588.0410	277.8831
4.2.07	389,487	244.8919	182028.0351	282.3417

Lena is then compared in Table 10 between our plan and other plans. It is clear that our system produces far less data than other systems, demonstrating our technique’s superior resilience to attacks based on pixel feature distribution.

**Table 10:** *χ*^2^ test comparison of our algorithm with other algorithms.

Algorithm	Proposed	EX1	EX2	Richman and Moorman (2000)	Liu et al. (2018)	Asgari-Chenaghlu et al. (2019)
*χ* ^2^	239.8008	21630.2343	261.5618	254.18	244.9922	262.054

## 5 Conclusion

The present color image encryption techniques either encrypt each of the three planes independently or they include repetitive processes that reduce the algorithm’s performance. To get beyond these problems, the paper has introduced a novel 1D chaotic system. By utilizing the new 1D chaotic system and Dijkstra algorithm, we have proposed a new improved Dijkstra algorithm and an adaptive diffusion cross-plane color encryption technique. We propose an image pixel that can make full use of the pixels of different planes and can directly process the three color planes of the color image to complete the cross-plane scrambling. A unique cross-plane permutation strategy has been suggested to increase the encryption system’s security and effectiveness. In the process of chaotic scrambling using cross-planes, we make great use of the relationship between different planar pixels, which makes the pixels very shuffled in order, pixels can appear at arbitrary coordinates on any plane, making it disrupting correlation between adjacent pixels and more difficult to predict pixel positions. Adaptive diffusion based on plane distribution utilizes the method of cross-plane diffusion, where any change in pixel values will result in a significant change in a large number of subsequent pixel values. According to the simulation results and security analysis in Chapter 4, it shows that our solution complies with various security standards, and most of the test indicators show that our solution is higher than the current popular image encryption schemes, it has been found to have stronger robustness and higher security. In this paper, differential attack experiment and resistance to data loss and noise simulated attack test are used respectively, and the experimental results show that our scheme is used that protects against attacks using specific plaintext and known plaintext, and compared to other schemes, our NPCR value is higher than other schemes, and the UACI value meets the safety standards. The original image is used to generate SHA-384 and a new chaotic system to compose the key, and the keyspace analysis shows that the keyspace size meets the security standards. The suggested approach has been demonstrated by simulation and security analysis to be successful, indicating that its security can render many attack schemes ineffective.

The proposed encryption technique avoids repeatedly encrypting the same areas of the image by making greater use of the correlation between pixels in distinct planes to encrypt the image just once. The improved Dijkstra algorithm used in this paper is a point-to-point encryption scheme. It avoids repeatedly encrypting the same areas of the image by making greater use of the correlation between pixels in distinct planes to encrypt the image just once. No new pixels are generated during the encryption process, and no pixels are lost, ensuring that the decrypted image is lossless. Color medical image is a special kind of RGB image, which has high privacy, and ciphertext security is related to the privacy and security of patients, Our scheme have been tested to the safety standards of Histogram Analysis, information entropy, analysis of adjacent pixel correlation, floating frequency test, image autocorrelation test, and *χ*^2^ test, data analysis has shown that our protocols meet safety standards and protect patient privacy. However, currently, this scheme is only applicable to RGB images since only the position relationship between the three planes of the color image is considered in the design, the encryption scheme of single-channel or multi-channel image is not considered and is not suitable for grayscale images or special images. Compared with other popular schemes, the encryption scheme proposed in this paper is normal in terms of speed and efficiency, but with the enlargement of image size, the number of chaotic iterations and the computation of the final position of the pixel are getting larger and larger, the time required by the proposed scheme is also increasing, and the time cost is higher when large-size image encryption is required, so it is not suitable for encryption scheme. In the future, we will attempt to develop schemes suitable for multichannel image encryption and remote image encryption.

## Data availability statement

Publicly available datasets were analyzed in this study. This data can be found here: (https://sipi.usc.edu/database/database.php).

## Author contributions

PH: Conceptualization, Data curation, Investigation, Methodology, Writing – original draft. YW: Conceptualization, Data curation, Formal analysis, Methodology, Writing – original draft. ZS: Conceptualization, Formal analysis, Funding acquisition, Resources, Supervision, Writing – review & editing. PZ: Resources, Validation, Writing – review & editing.
